# Improved specificity of a probe-based real-time PCR for the detection of *Dirofilaria immitis* in canine blood

**DOI:** 10.1186/s13071-026-07323-2

**Published:** 2026-08-20

**Authors:** Maureen A. Kelly, Tiana L. Sanders, Alexa Starnes, Emily Orr, Joe L. Luksovsky, Caroline Sobotyk, Hassan Hakimi, Pabasara Weerarathne, Christine M. Budke, Guilherme G. Verocai

**Affiliations:** 1 Department of Veterinary Pathobiology, College of Veterinary Medicine and Biomedical Sciences, Texas A&M University, College Station, TX 77843 USA; 2 Department of Pathobiology, School of Veterinary Medicine, University of Pennsylvania, Philadelphia, PA 19104 USA; 3 Department of Diagnostic Medicine and Pathobiology, College of Veterinary Medicine, Kansas State University, Manhattan, KS 66506 USA; 4 Department of Veterinary Integrative Biosciences, College of Veterinary Medicine and Biomedical Sciences, Texas A&M University, College Station, TX 77843 USA

**Keywords:** Antigen detection, Canine vector-borne diseases, Dogs, *Dirofilaria immitis*, Heartworm disease, Microfilariae detection test, Real-time PCR

## Abstract

**Background:**

*Dirofilaria immitis* is a vector-borne filarioid nematode distributed worldwide and endemic across most of North America. In dogs, it is considered the most clinically significant parasite and a known cause of acquired cardiomyopathy. Clinically, canine heartworm disease can manifest acutely as caval syndrome and chronically as heart failure, often resulting in death. Different types of diagnostic tests, such as antigen-detection and microfilariae-detection tests (MFDTs), including modified Knott’s test and molecular assays, may be used to diagnose *D. immitis* infection. Although some molecular tools, such as real-time PCR (qPCR), exhibit high accuracy, cross-reactivity with other *Dirofilaria* species found in companion animals remains a concern. Following our laboratory’s previously published protocol, cross-reactivity was found with *Dirofilaria striata* isolated from a subcutaneous nodule of a cat from Texas. The objectives of this study were to: (i) evaluate the performance of three newly designed probe-based qPCR assays for detecting *D. immitis* and (ii) assess overall detection with the newly designed protocols compared with the previously published protocol and other diagnostic tests.

**Methods:**

We designed three new probes using accessioned *D. immitis* cytochrome* c* subunit 1 sequences from various countries worldwide to increase reliability. These new probes were then tested with genomic DNA extracted from *D. immitis* and *D. striata.* Following this, we further assessed the performance of these newly designed probes and previously developed probe using 136 archival shelter dog samples previously found to be positive for *D. immitis* by at least one diagnostic test.

**Results:**

Out of the three probes tested, two (probe 2 and probe 3) specifically detected *D. immitis* and showed no cross-reactivity with *D. striata.* Probe 3 showed the highest prevalence of *D. immitis* among all qPCR assays (68.4%; *n* = 93/136 samples), followed by the original probe (66.9%; *n* = 91/136), with probe 2 (59.6%; *n* = 81/136) showing the lowest. Our analysis of agreement between probes using Cohen’s kappa (*κ*) statistic indicated almost perfect agreement between probe 3 and the previously published probe (*κ* = 0.86). The shelter dog samples were also evaluated using other heartworm diagnostic methods, including the modified Knott’s test, which was positive for 61.0% (*n* = 83/136) of the positive samples, and the previously published qPCR, which was positive for 66.9% (*n* = 91/136) of the samples. All samples were also tested in parallel with a commercially available antigen detection test (DiroCHEK®; Zoetis Inc., Parsippany, NJ, USA), yielding 80.1% positivity pre-heat treatment (*n* = 109/136) and 94.9% post-heat treatment (*n* = 129/136). Probe 3 paired with post-heat treatment achieved the highest positivity of the molecular diagnostic tests (97.1%; *n* = 132/136).

**Conclusions:**

This optimized probe-based qPCR assay (probe 3) provides an accurate and reliable method to detect *D. immitis* microfilariae in dogs and other carnivore hosts.

**Graphical Abstract:**

**Figure:**
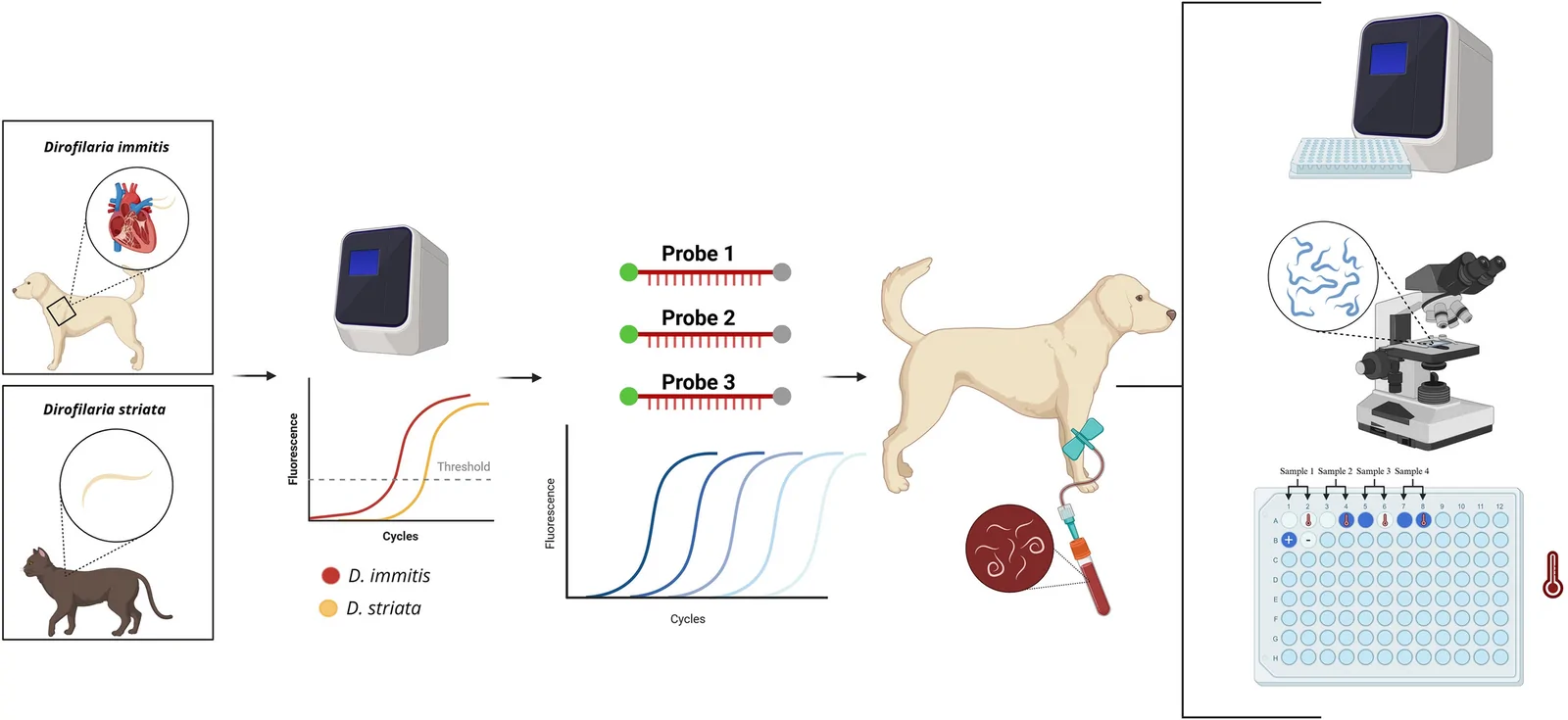

**Supplementary Information:**

The online version contains supplementary material available at 10.1186/s13071-026-07323-2.

## Background

Dirofilariasis, which is caused by filarial nematodes of the genus *Dirofilaria*, is a growing concern for veterinary and medical communities. These parasites can result in zoonotic infections and are found worldwide [1–5]. Both the Companion Animal Parasite Council (CAPC) and the American Heartworm Society (AHS) report a higher prevalence of dirofilariasis in the USA, making accurate diagnostics for identifying infected dogs essential [6, 7]. *Dirofilaria immitis* (Spirurida: Onchocercidae) is the causative agent of canine heartworm disease and is the most significant filarial nematode in veterinary medicine, primarily infecting dogs [1, 7, 8]. Heartworm is now well-established across nearly the entire world, particularly in tropical and subtropical regions, where its prevalence is higher [7–16]. This vector-borne pathogen is transmitted by over 20 mosquito genera [10, 17–20]. In the USA, mosquitoes known to vector *D. immitis* include species in the genera *Aedes*, *Anopheles* and *Culex* [14, 21–24]. Studies also show that many other animals are susceptible hosts, including cats, ferrets, coyotes and wolves [7, 8, 25–32]. Clinical signs of canine heartworm disease vary from subclinical or mild symptoms like coughing and activity intolerance to more severe clinical manifestations such as dyspnea, respiratory distress and heart failure, which can potentially lead to fatalities [7, 8, 10]. Humans are considered accidental hosts and can develop pulmonary dirofilariasis, characterized by a “coin lesion” within the pulmonary artery [3, 7, 8, 33–36].

The current recommendations for diagnosing *D. immitis* include performing two tests in parallel: a serology-based test and a microfilariae detection test (MFDT) [6, 7, 37]. Many commercially available serology tests target the antigen produced by the reproductive tract of adult females [7, 38–40] and are generally more sensitive than a MFDT in large-scale screening studies for detecting the status of heartworm infection in canines [16, 27, 41–45]. However, false-negative results can occur when circulating antigens bind the dog’s antibodies, forming immune complexes that hinder detection by standard serological assays [46–53]. Heat treatment has been a successful method of immune-complex dissociation (ICD) for heartworm antigen detection [46–48, 51–54], but it can cause cross-reactivity with other parasites such as *Dirofilaria repens*, *Angiostrongylus vasorum*, and *Spirocerca lupi*, which are known to infect dogs [55–58].

Microscopy-based techniques, such as direct smears, modified Knott's tests and microfilariae (mf) filtration tests, are commonly used as MFDTs [7]. Of these, a direct smear is the most easily applied technique in a clinical setting [7, 16, 37–39, 59]. Yet, many of these methods have disadvantages, including the need for extensive laboratory training to distinguish co-infection with multiple species, intensive labor input and an increased risk of false negatives, especially when mf may not be present in subclinical or early-stage infections [16, 38, 60]. The development of molecular diagnostics, such as conventional PCR (cPCR) and real-time PCR (qPCR), has significantly improved the detection of mf, especially in suspected infections, subclinical cases and large-scale epidemiological studies in dogs and cats [14, 16, 45, 53, 54, 60–63]. This highlights the need to develop highly sensitive and specific molecular techniques for rapid detection and accurate identification of *D. immitis* mf. Improved molecular tests can be easily integrated into screening studies for *D. immitis* and provide updated prevalence values in endemic and non-endemic regions.

It would be ideal to conduct large-scale screening studies in companion animals to assess the prevalence of h *D. immitis* in endemic and non-endemic regions. To achieve this, we must ensure that molecular assays are specific enough to distinguish *D. immitis* from other filarial nematodes that may be present in the blood of both hosts, such as *Dirofilaria striata.* Therefore, the objectives of this study were to first evaluate the sensitivity and specificity of three newly designed qPCR probes for detecting *D. immitis* and then to compare the overall detection of these new protocols alongside our previously published qPCR protocol [16] and other diagnostic tests commonly used for mf and antigen detection.

## Methods

### Optimization of qPCR

#### *Dirofilaria striata* sample collection

Filarial nematode fragments were recovered from a 3-year-old male domestic short-haired cat at the Texas A&M Veterinary Medicine Diagnostic Laboratory (College Station, TX, USA). The fragments were collected from a wound in the interscapular area and preserved in formalin before being sent to the Texas A&M University Parasitology Diagnostic Laboratory. Once received, morphological identification was not possible; therefore, genomic DNA was extracted using the QIAamp® DNA FFPE Tissue Kit (Qiagen, Hilden, Germany) according to the manufacturer’s instructions. Subsequently, DNA was screened with cPCR targeting the mitochondrial cytochrome *c* oxidase subunit 1 (*cox1*) gene fragment [61]. The amplified products were purified using the Cycle Pure E.Z.N.A.® Kit (Omega BioTek, Norcross, GA, USA) and then sent directly for Sanger sequencing (Eurofins, Louisville, KY, USA), yielding both forward and reverse sequences. After confirming *D. striata* via sequence alignment in MEGA X, it was submitted to GenBank (Accession no. PX315770) [64].

#### Design and development of three probes

Negron et al. [16] recently optimized a probe developed as an alternative to the modified Knott’s test [60]. In the present study, we tested the clinical sample described in the preceding section using the optimized protocol of these authors under standard cycling conditions [16]; the test results exhibited cross-reactivity, requiring further optimization. We then designed three TaqMan® probes using Primer Express software (Thermo Fisher Scientific Inc., Waltham, MA, USA) within the same targeted *cox1* region as the probe utilized in Negron et al. [16], which will be referred to hereafter as the “original probe”. Subsequently, we tested all four probes (i.e., original probe [16], probe 1, probe 2 and probe 3 [this study]) in combination with forward and reverse primers designed by Laidoudi et al. [60] (Table 1). Each probe/primer set was tested using DNA from a *D. immitis* adult female specimen and from *D. striata* adult fragments. Probes that did not cross-react were further assessed by standard curve analysis to determine the limit of detection (LOD) for DNA from adult specimens and mf-infected canine whole blood. This involved performing 10-fold dilutions using two *D. immitis* sample types: an adult female specimen (2600 pg/μl) and whole blood from an experimentally infected dog with high mf levels (12,000 pg/μl).

**Table 1:** Primers and probes designed for the amplification of the cytochrome* c* oxidase subunit 1 gene region of this study

Name	Sequence (5′- 3′)	References
*Primers*		
Fil.COI.749-F	CATCCTGAGGTTTATGTTATTATTTT	[60]
Fil.COI.914-R	CWGTATACATATGATGRCCYCA	[60]
*Probes*		
Original probe	6FAM-CGGTGTTTGGGATTGTTAGTG-MGB-NFQ	[60]
Probe 1	6FAM-GGTGTTTGGGATTGTTAGTGAA-MGB-NFQ	Current study
Probe 2	6FAM-CCAGACTAGTATG-MGB-NFQ	Current study
Probe 3	6FAM-TATTTGGATTGCTGTATTGGGG-MGB-NFQ	Current study

### Implementation of new probe-based qPCRs

#### Sample collection

To test the accuracy of the newly developed assays, we selected frozen, archival whole blood samples (*n* = 136) from domestic dogs (*Canis lupus familiaris*) collected between February 2020 and June 2023. All samples included in the study were collected from dogs in three shelters in Brazos County (*n* = 133) and Harris County (*n* = 3), Texas, USA. The collection method of these samples is described in Negron et al. [16]. The original qPCR probe, the modified Knott’s test and the DiroCHEK® canine heartworm test (Zoetis Inc., Parsippany, NJ, USA), both pre-ICD post-ICD, were evaluated for each blood sample tested in the study by Negron et al. [16]. Each sample tested positive for at least one test. In addition to the newly designed qPCR probes, the only diagnostic repeated on the selected samples was the original qPCR probe optimized by Negron et al. at standard cycling conditions [16].

#### Genomic DNA extraction and qPCR

Prior to DNA extraction, 200 μl of whole blood was placed in a thermomixer (37 °C) at 350 RPM overnight. Genomic DNA was extracted using the QIAamp® DNA Mini Blood Kit (Qiagen) following the manufacturer’s instructions. All extracted DNA was stored at − 20 °C until analysis.

All samples (*n* = 136) were screened for *D. immitis* using the original qPCR probe [16] along with the newly optimized probes. The assay target was a 166-bp fragment of the *cox1* region, initially designed by Laidoudi et al. [60]. The layout of each plate included duplicate samples for each probe/primer combination tested (Additional file 1: Figure S1). We also included positive and negative controls in single-well reactions, with duplicate no-template controls for each set. All reactions were performed in a 20-µl volume containing 3.5 µl of molecular-grade water, 0.5 µl (50 µM) of each primer, 0.5 µl (20 µM) of probe, 10 µl of 2 × TaqMan® Fast Advance Master Mix (Applied Biosystems, Thermo Fisher Scientific) and 5 µl of DNA template. All qPCR reactions were run on a QuantStudio™ 3 real-time PCR system (Applied Biosystems, Thermo Fisher Scientific) under the following standard cycling conditions: a denaturation step at 95 °C for 2 min, followed by 40 cycles of two-step PCR with denaturation at 95 °C for 1 s and annealing at 60 °C for 20 s, with a total run time of 43 min. The positive control consisted of DNA extracted from an adult *D. immitis* specimen, confirmed both morphologically and molecularly. In contrast, the negative control contained DNA extracted from whole blood collected from a dog negative for *D. immitis*, as confirmed by microscopy and molecular testing. The no-template control was nuclease-free molecular water. The qPCR results were analyzed using Design and Analysis 2 software to determine the presence or absence of *D. immitis* within each well (Applied Biosystems, Thermo Fisher Scientific).

### Data analysis

Statistical analyses were conducted using STATA® version 19.5 BE-Basic Edition (College Station, TX, USA). Descriptive statistics and analyses of positivity across all diagnostic tests were summarized in tables. The correlation between mf counts in each modified Knott’s test and the cycle threshold (Ct) values of each qPCR protocol was evaluated. Additionally, Cohen’s Kappa (*κ*) was calculated for each pair of diagnostic tests to assess the level of agreement between diagnostic tests [65–67]. The agreement levels were classified as follows: *κ* ≤ 0, no agreement; *κ* = 0.01–0.2, slight agreement; *κ* = 0.21–0.4, fair agreement; *κ* = 0.41–0.6, moderate agreement; *κ* = 0.61–0.8, substantial agreement; and *κ* = 0.81–1.0, almost perfect agreement [65–67].

We applied Cochran’s Q test to evaluate whether there were differences in the proportions of positivity across the six diagnostic tests (probe 2, probe 3, the original qPCR probe, the modified Knott’s test, DiroCHEK® pre-ICD and DiroCHEK® post-ICD). Probe 1 was not evaluated due to cross-reactivity. Subsequently, we performed Cochran’s Q test on all 15 combined diagnostic pairs and evaluated each set in parallel. Each test was followed by a post hoc analysis using McNemar’s test to compare diagnostic tests pairwise, with the Bonferroni correction applied to control for multiple comparisons. A *p*-value of ≤ 0.05 was considered statistically significant unless otherwise adjusted.

## Results

### Probe-based qPCR optimization

**Fig. 1:**
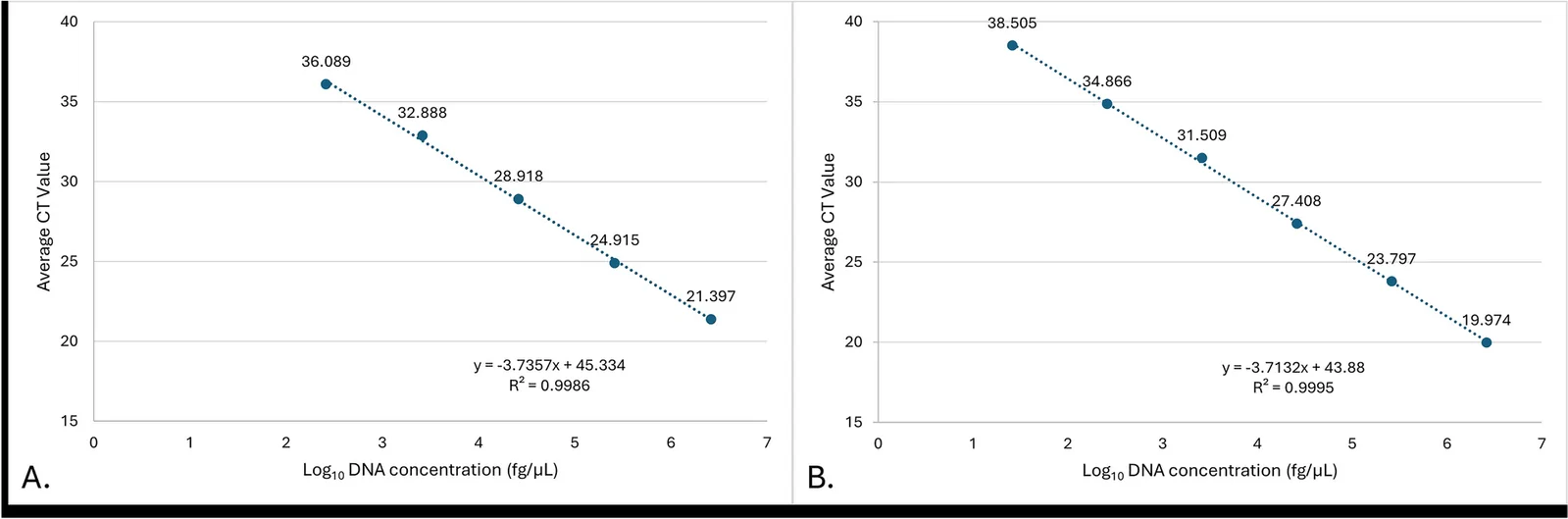
**A** Standard curve of real-time PCR (qPCR) for the detection of *Dirofilaria immitis* for probe 2. This was created by plotting the average cycle threshold (Ct) values of triplicates using a 10-fold serial dilution of adult specimen genomic DNA. **B** Standard curve of qPCR for the detection of *D. immitis* in probe 3. This was created by plotting the average Ct values of triplicates using a 10-fold serial dilution of adult specimen genomic DNA

**Fig. 2:**
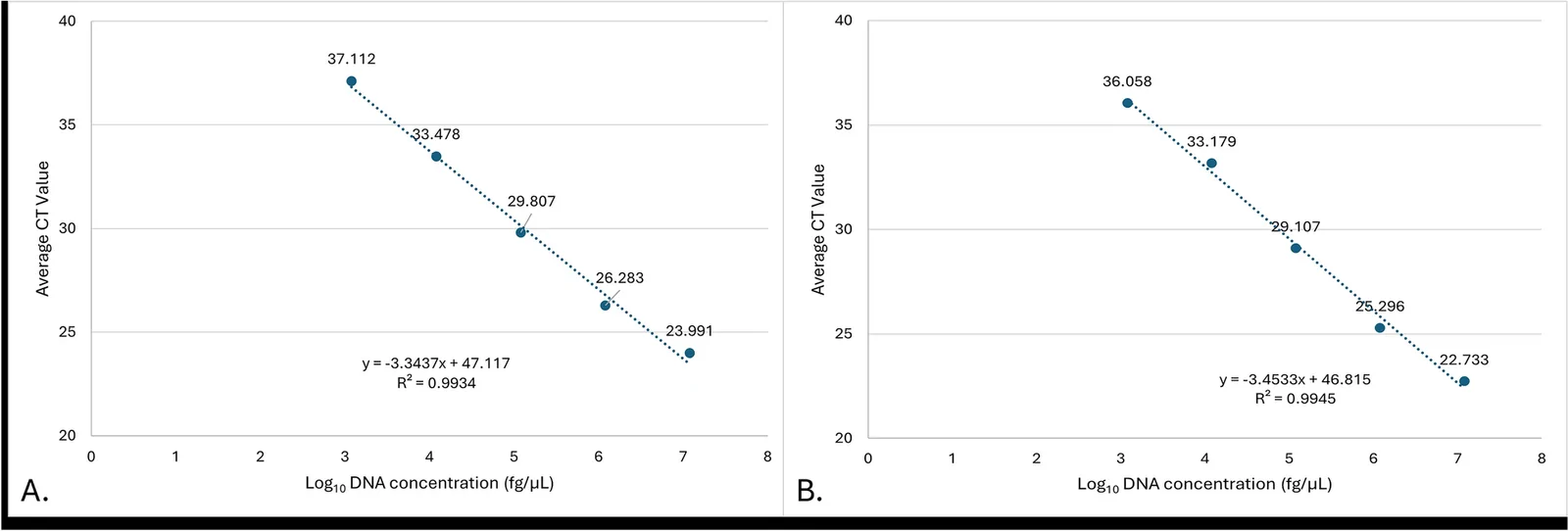
**A** Standard curve of real-time PCR (qPCR) for the detection of *Dirofilaria immitis* microfilariae in whole blood of experimentally infected dog samples using probe 2. This was created by plotting the average cycle threshold (Ct) values from triplicate samples using a 10-fold serial dilution of the microfilariae genomic DNA within each sample. **B** Standard curve of qPCR for the detection of *D. immitis* microfilariae in whole blood of experimentally infected dog sample using probe 3. This was created by plotting the average Ct values from triplicate samples using a 10-fold serial dilution of the microfilariae genomic DNA within each sample

Following preliminary testing, the original qPCR protocol [16] cross-reacted with the *D. striata* specimen identified above. This led to the design of three new probes that were compatible with the same primer set. We then tested all three probes in duplicate and found that probe 1 detected both *D. immitis* and *D. striata*, whereas probes 2 and 3 detected only *D. immitis*. Following a 10-fold standard curve, the LOD of probe 2 and probe 3 was 1300 fg and 130 fg for adult specimens, respectively (Fig. 1). In contrast, in whole blood infected with *D. immitis* mf, the LOD was 6000 fg for both probe 2 and probe 3 (Fig. 2). Probe 2 and probe 3 were selected to be utilized in the second objective of this study.

### Application of the selected probe-based qPCRs

The results of each diagnostic test, individually and paired (positive in test 1, test 2 or both), are summarized in Table 2. Following the screening of previously tested whole blood DNA (*n* = 136 samples), the highest positivity was in probe 3 (*n* = 93/136; 68.4%), followed by the original probe [16] (*n* = 91/136; 66.9%), with the lowest positivity in probe 2 (*n* = 81/136; 59.6%). No significant correlation was found between mf counts and Ct values for any probes (Additional file 2: Figure S2; Additional file 3: Figure S3; Additional file 4: Figure S4). The mf of *D. immitis* ranged from 1 to 88,803 per milliliter, with an average of 15,698 mf/ml. Regarding antigen detection, we found the highest individual rate in the DiroCHEK® post-ICD test with 94.9% positivity (*n* = 129/136). In the paired tests, we found that probe 3/DiroCHEK® post-ICD had the highest positivity (*n* = 132/136; 97.1%), followed by probe 2/DiroCHEK® post-ICD (*n* = 131/136; 86.3%). The lowest overall positivity was probe 2/DiroCHEK® pre-ICD (*n* = 113/136; 83.1%).

**Table 2:** *Dirofilaria immitis* positivity of all heartworm diagnostic tests performed both individually and paired within shelter dogs (*n* = 136) from Texas

Diagnostic test(s)	Number of *Dirofilaria immitis*-positive dogs (%)	95% confidence interval
*Individual test*		
MK	83 (61.0)	52.2–69.2
PO	91 (66.9)	58.3–71.3
P2	81 (59.6)	50.8–67.8
P3	93 (68.4)	59.8–76.0
DiroCHEK® pre-ICD	109 (80.1)	72.4–86.4
DiroCHEK® post-ICD	129 (94.9)	89.6–97.9
*Paired tests*		
MK + DiroCHEK® pre-ICD	116 (85.3)	78.2–90.8
MK + DiroCHEK® post-ICD	135 (99.3)	86.0–100.0
PO + DiroCHEK® pre-ICD	114 (83.8)	76.5–89.6
PO + DiroCHEK® post-ICD	131 (96.3)	91.6–98.8
P2 + DiroCHEK® pre-ICD	113 (83.1)	75.7–89.0
P2 + DiroCHEK® post-ICD	131 (96.3)	91.6–98.8
P3 + DiroCHEK® pre-ICD	115 (84.6)	77.4–90.2
P3 + DiroCHEK® post-ICD	132 (97.1)	92.6–99.2

*MK* Modified Knott’s test,* PO* original probe,* P2* probe #2 (this study),* P3* probe #3 (this study),* DiroCHEK® pre-ICD* DiroCHEK® test pre-immune-complex dissociation,* DiroCHEK® post-ICD* DiroCHEK® test post-immune-complex dissociation

In Table 3, Cohen’s kappa showed almost perfect agreement between MFDTs with the original probe/probe 3 pair (*κ* = 0.86), followed by the modified Knott’s test/probe 2 pair (*κ* = 0.84) and the original probe/probe 2 pair (*κ* = 0.84). We also found a substantial agreement between probe 2 and probe 3 (*κ* = 0.81), between the modified Knott’s test and original probe (*κ* = 0.68) and between the modified Knott’s test and probe 3 (*κ* = 0.67). We found a slight agreement when DiroCHEK® post-ICD was compared with all three molecular MFDTs—the original probe (*κ* = 0.11), probe 3 (*κ* = 0.07) and probe 2 (*κ* = 0.07)—and a poor agreement with the modified Knott’s test (− 0.06).

**Table 3:** Summary of the level of agreement between diagnostic tests for heartworm detection

Comparison of two tests (test 1/test 2)	a (+/+)	b (+/–)	c (–/+)	d (–/–)	Cohen’s kappa (*κ*)	Agreement interpretation
MK/PO	77	6	14	39	0.6822	Substantial
MK/P2	77	6	4	49	0.8465	Almost perfect
MK/P3	78	5	15	38	0.6799	Substantial
MK/pre-ICD	76	7	33	20	0.3215	Fair
MK/post-ICD	77	6	52	1	−0.0634	Poor
PO/P2	81	10	0	45	0.8428	Almost perfect
PO/P3	88	3	5	40	0.8656	Almost perfect
PO/pre-ICD	86	5	23	22	0.4827	Moderate
PO/post-ICD	89	2	40	5	0.1133	Slight
P2/P3	81	0	12	43	0.8102	Substantial
P2/pre-ICD	77	4	32	23	0.4016	Fair
P2/post-ICD	79	2	50	5	0.0770	Slight
P3/pre-ICD	87	6	22	21	0.4710	Moderate
P3/post-ICD	90	3	39	4	0.0784	Slight
Pre-ICD/post-ICD	108	1	21	6	0.2953	Fair

*MK* Modified Knott’s test,* PO* original probe,* P2* probe #2 (this study),* P3* probe #3 (this study),* pre-ICD* DiroCHEK^®^ test pre-immune-complex dissociation, *post-ICD* DiroCHEK^®^ test post-immune-complex dissociation

There was a significant difference in the Cochran’s Q test comparing proportions of positivity across the six diagnostic tests (*p* < 0.001) (Table 4). In the post-hoc analysis with a Bonferroni correction, differences remained significant for multiple diagnostic comparisons, including the modified Knott’s test/pre-ICD (*p* < 0.001) and the modified Knott's/post-ICD (*p* < 0.001) (Table 5). We also found significant differences between the original probe/probe 2 (*p* = 0.002) and between probe 2/probe 3 (*p* < 0.001). Finally, we observed a significant difference when each qPCR (original probe, probe 2, and probe 3) was evaluated with pre-ICD and post-ICD (Table 5).

**Table 4:** Cochran’s Q test results for comparing the proportions of heartworm positivity across six individual diagnostic tests

Comparison of individual methods	Q statistic *df* *p*-value
MK	117.0 5 < 0.001*
PO	
P2	
P3	
Pre-ICD	
Post-ICD	

*MK* Modified Knott’s test,* PO* original probe,* P2* probe #2 (this study),* P3* probe #3 (this study),* Pre-ICD* DiroCHEK^®^ test pre-immune-complex dissociation,* Post-ICD* DiroCHEK^®^ test post-immune-complex dissociation

*Significant relationships at *p* < 0.05

**Table 5:** Post-hoc analysis of the Cochran’s Q test using a McNemar’s test for individual tests

Post hoc analysis	*p*-value
MK/PO	0.115
MK/P2	0.075
MK/P3	0.041
MK/Pre-ICD	< 0.001*
MK/Post-ICD	< 0.001*
PO/P2	0.002*
PO/P3	0.726
PO/Pre-ICD	< 0.001*
PO/Post-ICD	< 0.001*
P2/P3	< 0.001*
P2/Pre-ICD	< 0.001*
P2/Post-ICD	< 0.001*
P3/Pre-ICD	0.003*
P3/Post-ICD	< 0.001*
Pre-ICD/Post-ICD	< 0.001*

*MK* Modified Knott’s test,* PO* original probe,* P2* probe #2 (this study),* P3* probe #3 (this study),* Pre-ICD* DiroCHEK^®^ test pre-immune-complex dissociation,* Post-ICD* DiroCHEK^®^ test post-immune-complex dissociation

*Significant after Bonferroni correction (α = 0.008)

For paired diagnostic tests, Cochran’s Q test revealed that all 15 combinations were statistically significant (*p* < 0.001) (Table 6). Post hoc McNemar tests indicated that molecular MFDTs (i.e., the original probe, probe 2, and probe 3) were statistically significant (*p* < 0.001) when paired with an antigen detection test (pre- or post-ICD), as opposed to pairing with a microscopy-based MFDT (i.e., modified Knott’s test) (Table 7). Among all probes compared in this study, probe 3 consistently showed a significant difference when paired with an antigen detection test, especially with post-ICD applied, indicating a substantial difference in detecting *D. immitis.*

**Table 6:** Cochran’s Q test results for comparing paired diagnostic tests for heartworm detection

Combination^a^	Q statistic *df* *p*-value
MK + PO (A)	340.519 14 < 0.001*
MK + P2 (B)	
MK + P3 ©)	
MK + Pre-ICD (D)	
MK + Post-ICD (E)	
PO + P2 (F)	
PO + P3 (G)	
PO + Pre-ICD (H)	
PO + Post-ICD (I)	
P2 + P3 (J)	
P2 + Pre-ICD (K)	
P2 + Post-ICD (L)	
P3 + Pre-ICD (M)	
P3 + Post-ICD (N)	
Pre-ICD + Post-ICD (O)	

*MK* Modified Knott’s test,* PO* original probe,* P2* probe #2 (this study),* P3* probe #3 (this study),* Pre-ICD* DiroCHEK® test pre-immune-complex dissociation,* Post-ICD* DiroCHEK® test post-immune-complex dissociation

*Significant relationships at *p* < 0.05

**Table 7:** Post-hoc analysis of the Cochran’s Q test using a McNemar’s test for paired heartworm diagnostic tests

Post hoc analysis of paired tests^a^	*p*-value
A/B	0.194
A/C	0.892
A/D	0.005
A/E	< 0.001**
A/F	0.431
A/G	0.893
A/H	0.013
A/I	< 0.001**
A/J	0.597
A/K	0.020
A/L	< 0.001**
A/M	0.008
A/N	< 0.001**
A/O	< 0.001**
B/C	0.152
B/D	< 0.001**
B/E	< 0.001**
B/F	0.610
B/G	0.2448
B/H	< 0.001**
B/I	< 0.001**
B/J	0.441
B/K	< 0.001**
B/L	< 0.001**
B/M	< 0.001**
B/N	< 0.001**
B/O	< 0.001**
C/D	0.007
C/E	< 0.001**
C/F	0.356
C/G	0.788
C/H	0.019
C/I	< 0.001**
C/J	0.507
C/K	0.029
C/L	< 0.001**
C/M	0.012
C/N	< 0.001**
C/O	< 0.001**
D/E	< 0.001**
D/F	< 0.001**
D/G	0.003
D/H	0.737
D/I	0.001**
D/J	< 0.001**
D/K	0.618
D/L	0.001**
D/M	0.865
D/N	< 0.001**
D/O	0.003
E/F	< 0.001**
E/G	< 0.001**
E/H	< 0.001**
E/I	0.098
E/J	< 0.001**
E/K	< 0.001**
E/L	0.098
E/M	< 0.001**
E/N	0.185
E/O	0.055
F/G	0.513
F/H	0.001**
F/I	< 0.001**
F/J	0.795
F/K	0.002**
F/L	< 0.001**
F/M	< 0.001**
F/N	< 0.001**
F/O	< 0.001**
G/H	0.009
G/I	< 0.001**
G/J	0.692
G/K	0.014
G/L	< 0.001**
G/M	0.005
G/N	< 0.001**
G/O	< 0.001**
H/I	< 0.001**
H/J	0.002**
H/K	0.870
H/L	< 0.001**
H/M	0.868
H/N	< 0.001**
H/O	0.001**
I/J	< 0.001**
I/K	< 0.001**
I/L	1
I/M	0.001**
I/N	0.734
I/O	0.758
J/K	0.004
J/L	< 0.001**
J/M	0.001**
J/N	< 0.001**
J/O	< 0.001**
K/L	< 0.001**
K/M	0.741
K/N	< 0.001**
K/O	< 0.001**
L/M	0.001**
L/N	0.734
L/O	0.758
M/N	< 0.001**
M/O	0.002**
N/O	0.519

**Significant after Bonferroni correction (α = 0.003)

^a^*A*: Modified Knott’s test + original probe, *B*: modified Knott’s test + probe 2, *C*: modified Knott’s test + probe 3, *D*: modified Knott’s test + DiroCHEK^®^ pre-immune-complex dissociation (pre-ICD), *E*: modified Knott’s test + DiroCHEK^®^ post-immune compex dissociation (post-ICD), *F*: original probe + probe 2, *G*: original probe + probe 3,* H*: original probe + DiroCHEK^®^ pre-ICD, *I*: original probe + DiroCHEK^®^ post-ICD, *J*: probe 2 + probe 3; *K*: probe 2 + DiroCHEK^®^ pre-ICD, *L*: probe 2 + DiroCHEK^®^ post-ICD,* M:* probe 3 + DiroCHEK^®^ pre-ICD; *N:* probe 3 + DiroCHEK^®^ post-ICD, *O*: DiroCHEK^®^ pre-ICD + DiroCHEK^®^ post-ICD

## Discussion

We designed a highly reliable and accurate probe-based qPCR protocol capable of detecting *D. immitis* within whole blood, serving as an additional confirmatory test for MFDTs. Based on current recommendations, to determine whether a dog is positive for *D. immitis*, two diagnostic modalities must be performed in parallel: an antigen-detection test and an MFDT [7, 8, 68]. This combined protocol increases diagnostic sensitivity and allows detection of infections that might otherwise be missed, including amicrofilaremic cases, infections with fewer than three females, infections with only juvenile females, male-only infections and cases with antibody-antigen complex formations [7, 8, 68]. In the present study, we further optimized a protocol initially designed by Laidoudi et al. [60] and subsequently refined with fast universal cycling parameters by Negron et al. [16]. Additionally, within Negron et al. [16], the original criteria for selecting shelter dog samples were the absence of preventive macrocyclic lactone use and an estimated age > 6 months. In the current study, we applied more specific inclusion criteria, requiring a positive result in at least one diagnostic test (modified Knott’s test, qPCR assay, DiroCHEK® pre-ICD, DiroCHEK® post-ICD) previously performed. After incorporating the selected, newly designed probe (probe 3), our modified assay detected two additional positives (*n* = 93/136) that were initially missed by the original protocol (*n* = 91/136) [16]. Utilizing probe 3, we found a lower detection threshold when compared to probe 2 for the detection of adult specimen DNA and mf DNA concentration (Fig. 1), whereas both probes were comparable in terms of the detection threshold for mf in canine blood, with probe 3 reporting an earlier Ct average (Fig. 2). This difference between these two sample types is likely due to the high concentration of the target DNA (*D. immitis*) of the selected *cox1* region. These findings highlight that the new assay is reliable for detecting mf in whole blood and can be utilized in future epidemiological investigations.

Compared with the original probe [16], probe 3 showed nearly perfect agreement as a diagnostic method. However, when the previously published protocol [16] was compared to probe 2, the results were statistically different. Overall, we found that probe 2 detected fewer positive samples than all of the other probes, suggesting the former is not highly reliable for this optimized protocol when combined with antigen detection as recommended [6–8]. In contrast, probe 3 aligns more closely with the original protocol [16] while providing higher accuracy for detecting *D. immitis* mf DNA in blood, especially in suspected, inconclusive cases, without cross-reacting with other filarial nematodes. This would ultimately lead to false-positive results and require costly treatment for the owner and unnecessary discomfort to the dog.

Upon closer examination of the sampled population, we identified a subset of dogs (*n* = 15) that tested negative with the modified Knott’s test. However, this same subset was positive via qPCR with probe 3 (*n* = 15/15) and was also positive under the previous qPCR protocol (*n* = 11/15) [16]. It is also worth noting that, among these 15 samples, the majority tested positive for antigens both pre-ICD (*n* = 13/15) and post-ICD (*n* = 14/15). These results highlight how inconclusive and discordant results may arise in practice, potentially delaying treatment decisions or leading to undiagnosed infections [7, 8]. Collectively, these findings suggest that incorporating DNA-based methods for detecting *D. immitis* mf is important, as they offer higher sensitivity and specificity, which are critical for accurate confirmation.

Currently, heat treatment is not widely recommended due to the risk of false positives when detecting circulating antigens produced by other parasites, such as *D. repens*, *A. vasorum* and *S. lupi* [55–58]. Additionally, new resources are constantly being developed and updated to aid in determining when to use heat treatment for ICD during annual *D. immitis* testing when an inconclusive result is obtained [42, 50, 69]. However, in the present study, the highest positive pairing of antigen and molecular-based MFDT was observed with probe 3/DiroCHEK® post-ICD (*n* = 132/136; 97.1%). This result led to further evaluation of potential cross-reactivity with these parasites, which are known to produce false positives. We could not, however, assess this qPCR protocol due to limited DNA availability; instead, we evaluated it using bioinformatics in MEGA X [64] to assess potential amplification in this DNA-based test (Additional file 5: Figure S5). Within Figure S5, we aligned and analyzed sequences of *D. immitis* as well as sequences of *D. striata*, *D. repens, S. lupi* and *A. vasorum* available in GenBank across several host types. Additionally, we included the sequences of each primer, the originally published probe [16] and both probe 2 and probe 3 to also assess the alignment with the targeted DNA. The aim of this analysis was to determine whether cross-reactivity could occur if DNA from these parasitic species were present in the blood during screening for *D. immitis*. Among the aligned sequences, *D. repens* is the most likely to cross-react as it is known to be co-endemic with *D. immitis* in regions such as Europe, Africa, Asia and, more recently, South America, but it has not yet been unequivocally confirmed in the USA [8, 45, 70–75]. While adults of *D. repens* are found in subcutaneous tissues, mf circulate in the blood, similar to *D. immitis*, and require similar confirmation techniques [7, 8, 33, 37, 57]. Conversely, *A. vasorum* and *S. lupi* produce first-stage larvae and eggs, respectively, which can be identified by fecal tests, such as the Baermann technique or fecal flotation [37]. Recently, *A. vasorum* was locally acquired in a dog in Oregon, and cases in wildlife such as red foxes, coyotes, and black bears have been identified [76, 77]. Similarly, *S. lupi* was documented in domestic dogs and wild carnivores in the USA, including Texas [78–81]. These biological and epidemiological factors further support the minimal likelihood of cross-reactivity with this qPCR assay.

The two additional positives would not have been found if we had not also identified cross-reactivity with *D. striata*, a filarioid nematode commonly reported in the blood of wild felids [82, 83]. *Dirofilaria striata* is a parasite documented in North America, but limited biological and epidemiological information are currently available. Its life-cycle is suspected to be similar to that of *D. immitis,* with mosquito species (*Anopheles quadrimaculatus*) required for transmission of the third-stage larvae [83]. This skin-dwelling filarial nematode has been associated with felids, specifically domestic cats in Florida [84] and bobcats in Louisiana and Texas [82, 83]. There has been only one documented case of *D. striata* mf found in a dog, which resided in Florida and had a travel history from Ohio [85]. Considering these factors, we improved the diagnostic sensitivity and specificity of this assay to avoid cross-reactivity with *D. striata*, enabling its potential use in large-scale epidemiological studies to determine the prevalence of *D. immitis* in both dogs, cats and wild reservoir hosts.

In a clinical setting, molecular assays, such as conventional and qPCR, may not be easily integrated, as each technique requires adequate personnel training, specialized reagents and equipment. These requirements can be costly for clinics with limited funding sources and staffing difficulties. Therefore, this qPCR assay would be ideal for confirmatory testing when inconclusive results are obtained, and it can be performed by reference and diagnostic laboratories as a “send-out” option. Molecular assays can also be used to test *D. immitis* in other susceptible hosts, such as cats, wild animals and marine life [26, 30, 86–91]. These hosts may play a role in transmitting the infection to surrounding definitive hosts, especially in high-risk areas [92, 93]. The detection of mf is not as easily confirmed in cats as in dogs; it is suspected that the cat’s immune system can control *D. immitis* mf and that these are unlikely to be detected using the same diagnostics as in dogs [8, 28, 94, 95]. This aspect was not evaluated in the current study because we did not have mf-positive blood from a cat during the optimization process. However, if this protocol were to be used and amplification was subsequently detected, it is crucial to follow the AHS feline guidelines [94]. These guidelines recommend that additional testing, such as antigen detection with heat treatment and antibody detection, be performed to confirm heartworm infection rather than treating the result as a false-positive in a cat or suspecting a *D. striata* infection [8, 32, 54, 94–98, 99]. Another approach to incorporating this assay is in large-scale studies, where it can be combined with commercially available serology tests to perform antigen and MFDT testing in parallel [13, 14, 16, 27, 45, 94, 97]. This would allow the use of optimized, accurate MFDT in combination with a serology-based test, thereby increasing the likelihood of detecting low-level *D. immitis* infections.

Molecular assays, such as probe-based qPCRs, are ideal for providing cost-effective, low-intensity, rapid results while facilitating easy implementation for inconclusive cases in reference laboratory facilities and large-scale research studies [16, 30, 45, 60, 63]. As highlighted in our study, there may also be disadvantages, including the potential for cross-reaction with other closely related nematode species. However, this can be avoided by incorporating in silico bioinformatics, combined with benchtop testing of the DNA from other filarioid nematodes, which enables real-time testing for accuracy and reliability, depending on the availability of DNA. As with microscopy-based techniques, morphological characterization of mf requires intensive training and extensive assessment. This is neither ideal nor possible within a clinical setting. An advantage of using molecular assays is the ability to include other molecular markers, such as *Wolbachia*, which enables the potential detection of *D. immitis* and co-infecting species (e.g., *Acanthocheilonema reconditum* and *D. repens*) [60, 100]. This will, in turn, require less sample collection, while providing results for other companion animal parasites that are less pathogenic but can cause health issues. Overall, we have provided an optimized protocol that can be used as an additional confirmatory MFDT and implemented in large-scale studies of both dogs and cats in combination with commonly performed serological-based tests.

## Conclusions

In the present study, we improved a probe-based qPCR method previously published by our laboratory. We found that increased accuracy enabled the detection of two additional positives that were previously unreported, while also limiting cross-reactivity with *D. striata*, a closely related filarioid nematode. This protocol presents an additional MFDT and a confirmatory test for cases in which viable options are available in subclinical or inconclusive routine diagnostics and can be easily implemented in large-scale screening studies.

## Supplementary Information

**Additional file 1: Figure S1.** An outline of what was included for each qPCR plate, including the originally published probe and the newly designed probes.

**Additional file 2: Figure S2. ** Correlation between the original probe qPCR Ct values and the number of microfilariae in blood.

**Additional file 3: Figure S3.** Correlation between the probe two qPCR Ct values and the number of microfilariae in blood.

**Additional file 4: Figure S4. **Correlation between the probe three qPCR Ct values and the number of microfilariae in blood.

**Additional file 5: Figure S5.** A visual representation of the newly designed protocols, probe 2 and probe 3, along with the original probe, targeting the cytochrome c oxidase subunit 1 for *Dirofilaria immitis* from various hosts. Also included are sequences of *Dirofilaria repens*, *Spirocerca lupi*, and *Angiostrongylus vasorum* to highlight bioinformatic differences.

## Data Availability

Data supporting the main objectives and conclusions are presented in the manuscript and supplementary files.

## Abbreviations

*cox1*: Cytochrome *c* oxidase subunit 1 gene fragment
cPCR: Conventional PCR
ICD: Immune-complex dissociation
mf: Microfilariae
MFDT: Microfilariae detection test
qPCR: Real-time PCR
